# Accuracy of Video Otoscopy in Predicting the Presence of Middle Ear Effusion in Children Compared to Tympanometry: A Diagnostic Study

**DOI:** 10.7759/cureus.73098

**Published:** 2024-11-05

**Authors:** Deema Othman, Masa Alashkar, Mohamad A Bitar

**Affiliations:** 1 College of Medicine, Mohammed Bin Rashid University of Medicine and Health Sciences, Dubai Health, Dubai, ARE; 2 Pediatric Otolaryngology, Al Jalila Children's Specialty Hospital, Dubai Health, Dubai, ARE

**Keywords:** middle ear effusion, otoscopy, speech delay, tympanic membrane, tympanometry

## Abstract

Importance: Middle ear effusion (MEE) is the primary cause of conductive hearing impairment among children, predominantly occurring up to the age of two years. The gold standard for detecting MEE is tympanometry (Grayson-Stadler, Eden Prairie, Minnesota). This study explores a less costly alternative, the video otoscope (Inventis S.R.L, Padova, Italy).

Objective: The primary objective is to compare video otoscopy with tympanometry in terms of its ability to diagnose MEE. The secondary objective is to explore the prevalence of MEE in special populations.

Methods: We conducted a retrospective diagnostic study that included patients aged 0-18 years who visited the pediatric otolaryngology clinic for suspected MEE over a two-year period. Clinical presentation, otoscopy findings, and tympanometry results were reviewed. The data were analyzed using IBM SPSS Statistics for Windows, Version 24 (Released 2016; IBM Corp., Armonk, New York). The significance of the results was assessed using the chi-squared test.

Results: We included 337 patients with a mean age of 5.1 years (standard deviation = 2.68); 967 tympanometry tests were available for comparison with the corresponding ears. Validity tests showed that the sensitivity of video otoscopy was 79.5%, the specificity was 56.9%, the positive predictive value was 89.6%, and the negative predictive value was 37.4%. The overall accuracy was 75.5%. These results were statistically significant.

Conclusion: Video otoscopy was capable of diagnosing MEE in children 89.6% of the time. However, tympanometry is still needed in specific conditions, such as narrow ear canals, dull tympanic membranes, and clear tympanic membranes in patients with decreased hearing, a history of ear infections, or speech delay.

## Introduction

Middle ear effusion (MEE) is a build-up of fluid in the space behind the eardrum, resulting from dysfunction in the Eustachian tube or the mucociliary system. It is the most common cause of conductive hearing loss in children, primarily occurring around the age of two. The incidence of MEE decreases with age, from 40% at two years to 1.4% at 11 years [1]. Incidence rates vary by geography and race; for example, a study by Holmquist et al. showed that Kuwaiti nationals are more affected than non-Kuwaitis [2].

MEE is typically assessed clinically using an otoscope. Smith noted that the first otoscope was introduced in 1830 by Jean-Pierre Bonnafont, who believed that the ear canal could be better visualized by shining light into the ear with a mirror [3]. Later, a handheld otoscope was invented, improving the visualization and assessment of the middle ear [4]. Recently, a more advanced otoscope, the video otoscope (Inventis S.R.L, Padova, Italy), became available in our clinic in 2016.

The video otoscope enables detailed visualization of the ear canal, eardrum, and ossicles through magnification, allowing fluid buildup, ear infections, foreign bodies, otitis media, and other abnormalities to be detected more easily than with a conventional otoscope [5]. However, otoscopy or video otoscopy does not always adequately assess middle ear pathology due to narrow canals, uncooperative patients, or dullness of the tympanic membrane (TM). In these cases, patients are referred for tympanometry (Grayson-Stadler, Eden Prairie, Minnesota) for a more objective evaluation of middle ear status, including MEE.

Tympanometry records TM movement in response to pressure variations, helping to determine the presence of any fluid in the middle ear or any ossicular dysfunction [6]. While video otoscopy provides valuable detail, it remains operator-dependent and subjective. Tympanometry, on the other hand, is regarded as an objective test when performed correctly. The correlation between findings on video otoscopy and tympanometry has been previously studied, primarily in cases of acute middle ear disease.

A study by Ting et al. reviewed 1,951 infected ears from patients aged 0.5 to 76 years between 2005 and 2014, comparing handheld otoscopy coupled with video otoscopy to tympanometry in these cases [7].

A recent prospective study by Aftab et al. compared oto-endoscopy with tympanometry in 75 acutely ill patients, aged 5 to 18 years, who were suspected of having acute middle ear pathology [8].

To our knowledge, video otoscopy has not been compared with tympanometry in detecting chronic MEE in children. Therefore, we conducted a study to assess the accuracy of video otoscopy in detecting chronic MEE in children presenting to a pediatric otolaryngology clinic, using tympanometry as the objective tool for this comparison.

## Materials and methods

We conducted a retrospective diagnostic study at Al Jalila Children’s Hospital (AJCH), including patients aged 0-18 years who visited the pediatric otolaryngology clinic between January 2019 and December 2020 for suspected persistent MEE. All patients were assessed by a single physician (senior author - pediatric otolaryngologist). Our inclusion criteria were suspected MEE, available tympanometry during the assessment visit, and examination with a video otoscope. Patients were selected through consecutive sampling. Exclusion criteria included acute ear infection and TM perforation at the time of assessment.

Ethical approval was granted by the Mohammed Bin Rashid University of Medicine and Health Sciences Institutional Review Board (IRB-2021-24). No funding was required for the study.

**Figure 1:**
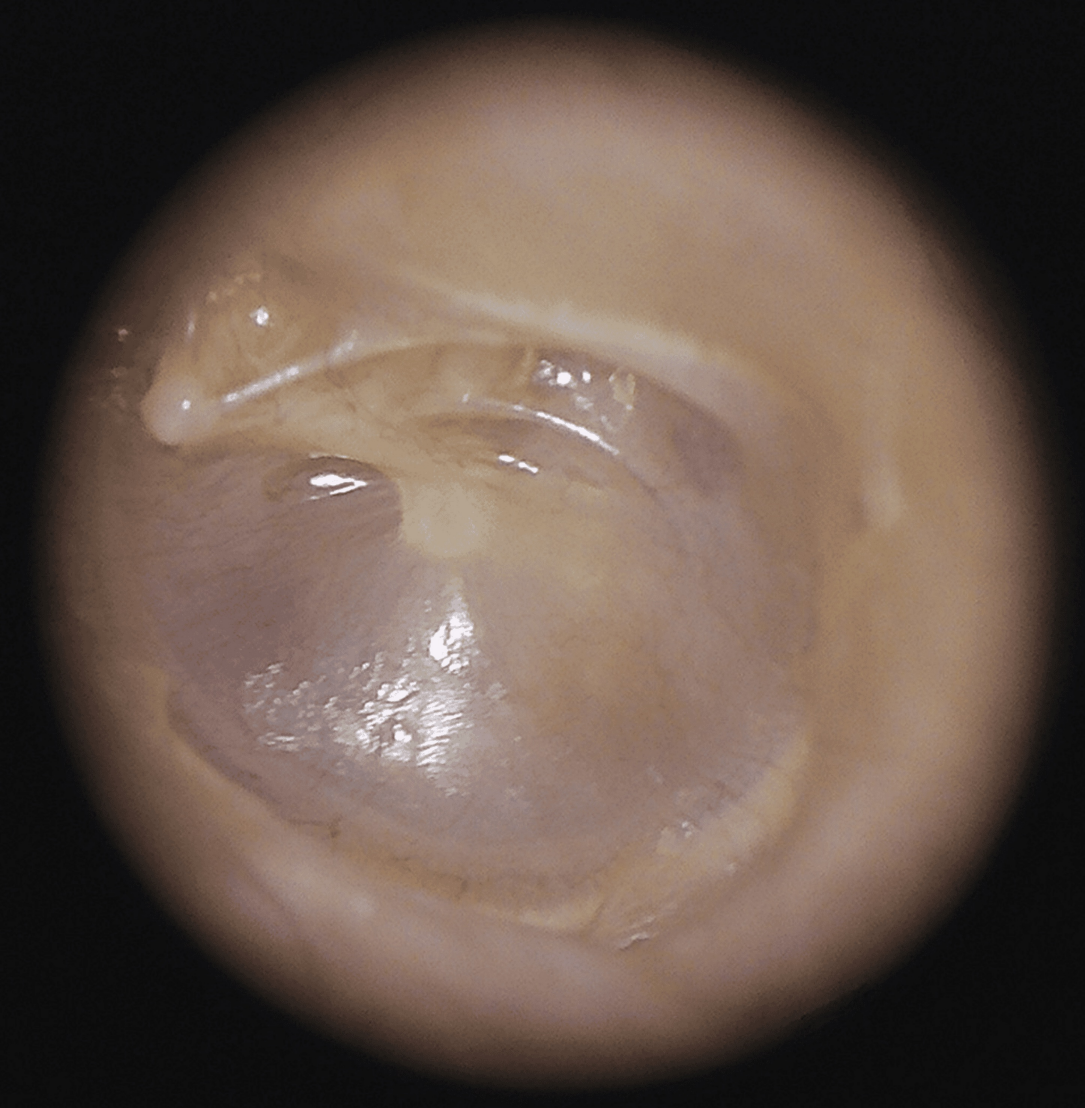
Magnified view of tympanic membrane as visualized by our video otoscope

We collected data on patient age and gender. We reviewed the clinical presentation, including speech delay, history of recurrent otitis media (OM), suspected hearing loss, or presence of a syndrome. We documented the details of the physical examination, noting any ear canal deformity, TM retraction, membrane dullness, and presence or suspicion of MEE. "Suspected MEE" refers to cases where the examiner believes there may be effusion due to discoloration of the drum or an air-fluid level but cannot confirm the diagnosis with certainty. Ear examinations were performed using a video otoscope (Inventis S.R.L, Padova, Italy) to visualize the tympanic membrane, as shown in Figure 1.

The findings on examination were classified under the following categories: MEE, suspected MEE, dull TM, retracted TM, narrow canal, and clear TM. Video otoscopy findings were considered positive if MEE, suspected MEE, or retracted TM were noted. A negative video otoscopy indicated a clear TM. Narrow canals (where the TM could not be well visualized) and dull TMs were categorized as indeterminate video otoscopy findings.

**Figure 2:**
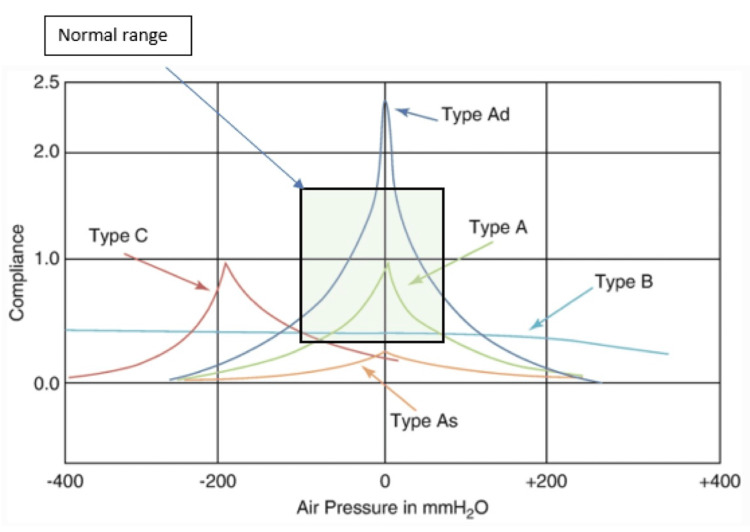
Tympanogram demonstrating the various curves reported on tympanometry

After the ear examination, patients were sent for tympanometry to be conducted during the same visit. This test was performed by an audiologist who was blinded to the patient's history and physical examination findings. The tympanometer is a device that sends sound waves (Hertz) at varying air pressures (decapascals) and measures the sound reflection from the TM. The test produces a tympanogram (Figure 2), which is a graphical representation of TM compliance (cm³ or mL) plotted against the different air pressures. In addition, it determines the external ear canal volume (cm³/mL) and the stapedial reflex [9-13].

Tympanometry results were documented and divided into positive and negative findings. Positive findings included As, B, C, and Cs curves, along with absent or elevated reflexes. Negative findings included A and Ad curves with present reflexes. Data were collected directly from patients’ files in AJCH’s database and transferred to an Excel datasheet.

The data were analyzed using IBM SPSS Statistics for Windows, Version 24 (Released 2016; IBM Corp., Armonk, New York). The chi-squared test was used to assess the significance of all results. Age was analyzed using mean, standard deviation, and a distribution curve. For our validity tests, we excluded indeterminate findings of video otoscopy (narrow canal and dull TM) to form a 2x2 table and calculate the sensitivity, specificity, positive predictive value, and negative predictive value of the index test [14]. We also calculated the accuracy of the index test.

Cases with positive video otoscopy but negative tympanometry were isolated for further analysis. The frequency of each positive video otoscopy finding was assessed to determine which finding was the most misleading in this group of patients.

Cases where video otoscopy was negative and tympanometry was positive were also analyzed. The patient’s history in this group was investigated for findings that raised the physician’s suspicion of MEE (e.g., speech delay, decreased hearing).

We grouped cases where both video otoscopy and tympanometry were positive and analyzed the reliability of each positive finding on video otoscopy (e.g., MEE, retracted TM). Down syndrome cases were then grouped separately to examine the prevalence of middle ear pathology, particularly as narrow canals are common in these patients [15,16]. Dull TM was not classified as a positive or negative finding on video otoscopy; it was analyzed with respect to tympanometry findings.

## Results

**Figure 3:**
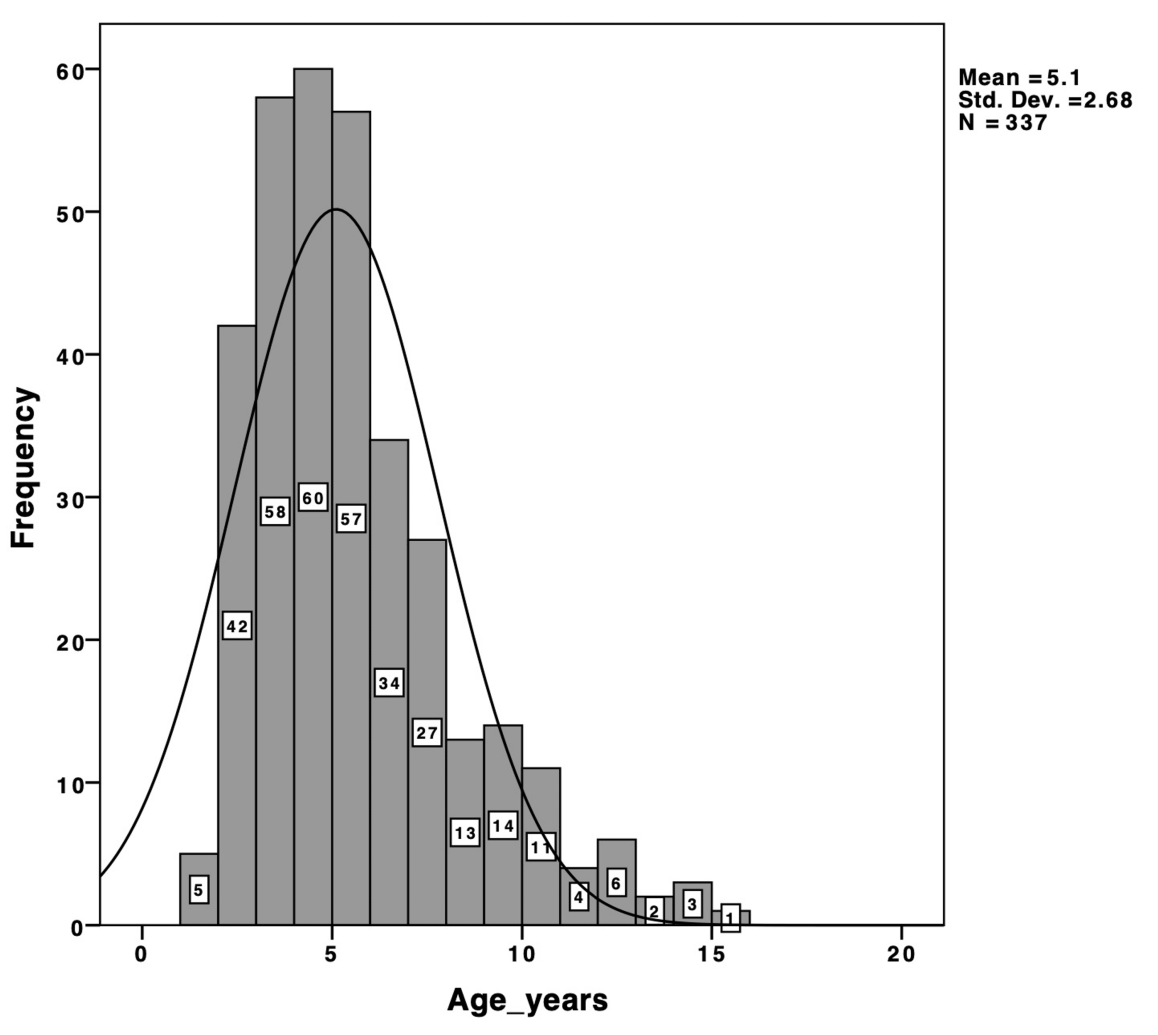
Age distribution of the 337 patients included in our study

We included 337 patients with a mean age of 5.1 years (standard deviation = 2.68); 76% of them were younger than seven years (Figure 3). Of these, 191 (56.7%) were male and 146 (43.3%) were female. A total of 967 tympanometry tests were available for comparison with the corresponding ears. Forty patients had Down syndrome and underwent 156 tympanometry tests. The patients' history is summarized in Table 1.

**Table 1:** History of patients referred to the pediatric otolaryngology clinic for suspected persistent middle ear effusion MEE: middle ear effusion, OM: otitis media.

Variables	Number of ears
History of MEE	349
Decreased hearing	145
History of recurrent OM	122
Speech delay	106
Others	21

**Figure 4:**
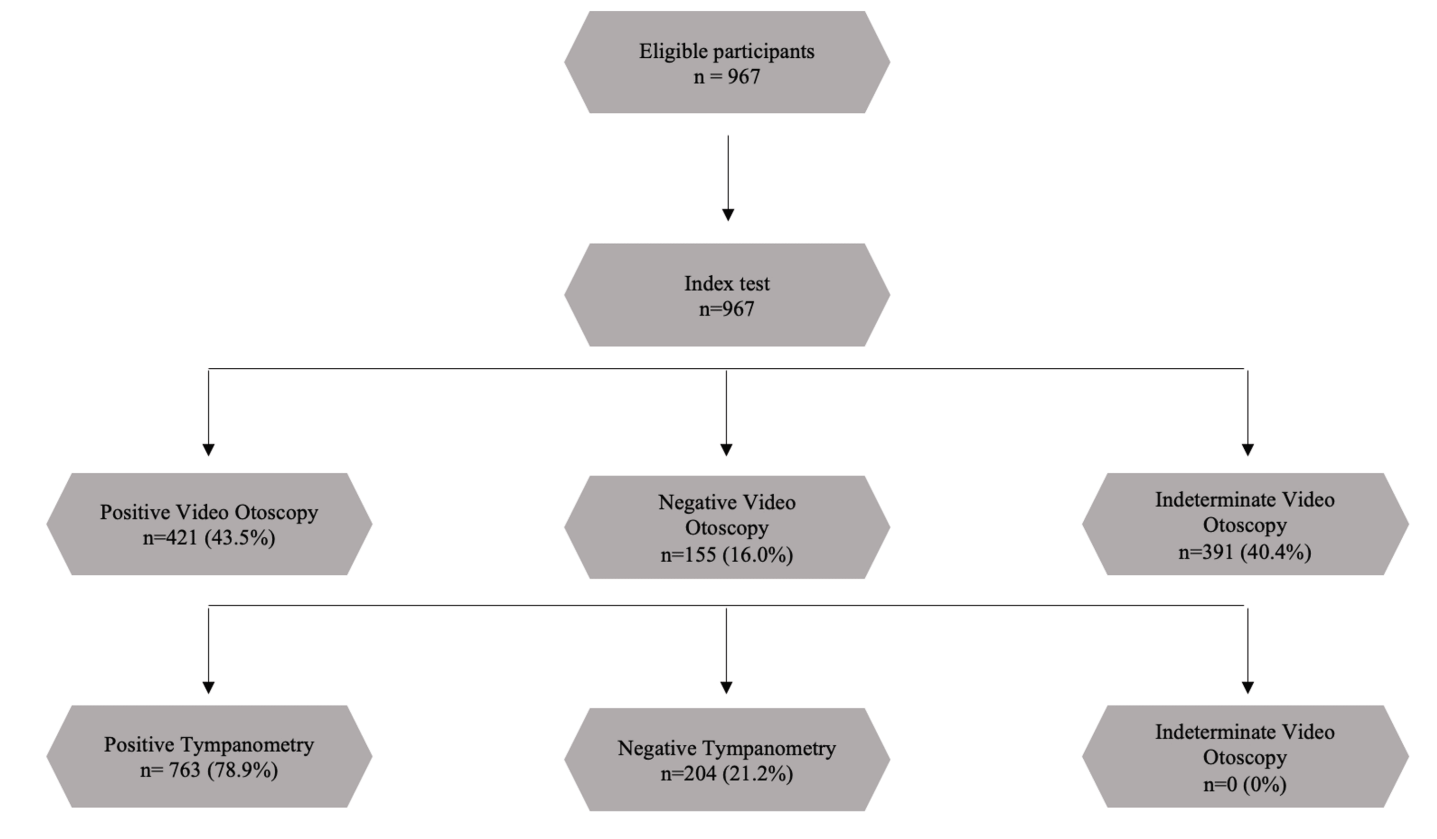
Flow of participants

Ear examination using the video otoscope was considered positive in 421 (43.5%) ears, negative in 155 (16.0%) ears, and indeterminate in 391 (40.4%) ears. The flow of participants is shown in Figure 4. Among the positive findings, 244 were classified as MEE, 141 as suspected MEE, and 107 as retracted TM (71 of which were combined with other positive findings). Among the indeterminate findings, 358 were dull TM, and 92 were narrow canal (59 of which also had dull TM).

The corresponding tympanometry was positive in 763 (78.9%) ears and negative in 204 (21.1%) ears. Among the positive tympanometry results, 338 were B curves, 163 were C curves, 71 were As curves, and 49 were Cs curves; 126 had an A curve, and 16 had an Ad curve, with either absent or elevated reflexes. Specifically, positive tympanometry results showed absent reflexes in 305 cases, elevated reflexes in 121, and present reflexes in 41, while 296 did not have tested reflexes. Among the negative tympanometry findings, 202 were A curves and two were Ad curves, with 166 showing present reflexes and 38 with reflexes not tested.

For validity analysis, indeterminate findings were excluded, leaving 576 samples. A comparison of tympanometry and video otoscopy findings is summarized in Table 2. From these data, the sensitivity of video otoscopy was 79.5%, and the specificity was 56.9% (Table 3).

**Table 2:** Comparison of video otoscopy and tympanometry results (n=576)

		Tympanometry		Total
		Positive	Negative	
Otoscopy	Positive	377	44	421
	Negative	97	58	155
Total		474	102	576

**Table 3:** Clinical performance of video otoscopy test as compared to tympanometry for detection of middle ear effusion in children P < 0.00001. CI: confidence interval, PPV: positive predictive value, NPV: negative predictive value.

Variables	Video otoscopy
Sensitivity (95% CI)	79.5% (75.6–83.1)
Specificity (95% CI)	56.9% (46.7–66.6)
PPV (95% CI)	89.6% (87.2–91.5)
NPV (95% CI)	37.4% (31.9–43.3)
Accuracy (95% CI)	75.5% (71.8–79.0)

We examined the 44 false positive findings on video otoscopy and found that 25/146 (17.1%) were classified as suspected MEE, 18/245 (7.3%) as MEE, and 9/108 (8.3%) as retracted TM. Among the 155 negative video otoscopy findings (clear TM), 97 had positive tympanometry results. Of these, 31/54 (57.4%) had a history of MEE, 27/35 (77.1%) had a history of decreased hearing, 18/23 (78.3%) had a history of recurrent OM, 15/22 (68.2%) had a history of speech delay, and 6/21 (28.6%) had a non-relevant history.

In the 377 ears in which both video otoscopy and tympanometry were positive, 226/244 (92.6%) were positive for MEE, 118/141 (83.7%) were positive for suspected MEE, and 98/107 (91.6%) were positive for retracted TM. A history of speech delay was associated with positive tympanometry in 76.4% of cases (81/106).

We further explored the dull TM finding present in 358 ears; 260 of these ears (72.6%) had positive tympanometry and 178 (49.7%) had a relevant positive history. Among these, 113 had a history of MEE, 48 had a history of recurrent OM, 43 had a history of decreased hearing, and 34 had speech delay. Of these 178 ears, 127 (71.3%) also had positive tympanometry.

On the other hand, of the 180 ears with a dull TM but no relevant positive history, 133 (73.9%) had positive tympanometry. A narrow canal with limited visualization of the TM was present in 92 ears, with 86 of them (93.5%) showing positive tympanometry.

In Down syndrome patients, narrow canals with limited TM visualization were detected in 89/156 examined ears (57.1%). Tympanometry was positive in most of these cases (147/156; 94.2%).

## Discussion

​​​​​Middle ear disease is a common problem in the pediatric age group, primarily including recurrent otitis media (OM), recurrent OM with effusion, and chronic OM with effusion. Most of our patients were younger than seven years (76%), putting them at risk for middle ear disorders [1,15].

In our pediatric otolaryngology clinic, many children are referred for middle ear-related issues, and most undergo tympanometry as part of the assessment. However, following our audiology unit's protocol, some children present with more general concerns, such as speech delay and decreased hearing, for which tympanometry is considered the first step in hearing evaluation.

During examination, video otoscopy was consistently used, providing an enlarged view of the TM and aiding in interpreting findings. However, MEE was not always visible, even with magnification, especially if the fluid was clear or the TM appeared quite dull. When fluid presence was clear, video otoscopy was reasonably reliable. However, if video otoscopy findings were negative, the reason for the patient’s visit (e.g., decreased hearing, recurrent OM, or speech delay) influenced the decision to perform tympanometry to rule out MEE.

A history of speech delay was an important factor to consider regardless of video otoscopy findings. The strong correlation between speech delay and a positive tympanometry result (76.4%) supports this approach. We recommend ordering tympanometry even if video otoscopy does not show abnormalities in a patient with speech delay, given the relatively high yield (68.2%).

In examining children, we often encounter narrow canals that make TM visualization difficult. In these cases, tympanometry is a highly reliable tool for revealing middle ear issues that may otherwise be missed. Down syndrome patients exemplify this context, as they are at high risk of developing MEE, which was reconfirmed in our study group (94.2%) [17].

Validating magnified otoscopy via tympanometry has been previously reported by Aftab et al., who studied 75 patients aged 5 to 18 years visiting the ear, nose, and throat outpatient department with signs and symptoms of acute otitis media and otitis media with effusion [8]. Not only did these patients present with acute symptoms, but the tool used was a video endoscope, which is somewhat invasive and challenging to use safely on children.

Another similar study was conducted by Ting et al., which included 900 patients aged 0.5 to 76 years with infected ears based on chief complaints and handheld otoscope examination. These patients were encouraged to follow up every three to five days at an outpatient clinic for observation using a video otoscope and tympanogram, and the results were compared [7]. This differs from our study, which excluded acute infections and was conducted purely in children.

The interpretation of findings on video otoscopy is subjective and operator-dependent; we tried to limit this impact by including readings from only one physician. However, we acknowledge that the results of this study might vary according to the physician's experience, technical skills, and training. Furthermore, it is important to note that the population enrolled in this study consists of patients referred to a specialty clinic, not a primary care setting. Despite this, our findings, including the need for tympanometry in cases with narrow canals, can be generalized to all settings, as a complete evaluation of the tympanic membrane is often challenging. The study was not limited to patients referred with MEE, to reflect what the physician typically encounters in the clinic; this allowed us to study various relevant clinical scenarios rather than focusing on a single pathology. This approach helped demonstrate the usefulness of combining a detailed clinical history with video otoscopy findings.

## Conclusions

This study demonstrated that video otoscopy can assist in diagnosing MEE in children. It is a cost-effective alternative to tympanometry for diagnosing MEE. If tympanometry is not readily available, video otoscopy can aid general practitioners, pediatricians, and otolaryngologists in detecting MEE in children. However, video otoscopy cannot replace tympanometry in ruling out MEE in specific conditions, such as narrow ear canals, dull tympanic membranes, and clear tympanic membranes in patients with decreased hearing, a history of ear infections, or speech delay.
